# Evaluation of the cancer chemopreventive efficacy of rice bran in genetic mouse models of breast, prostate and intestinal carcinogenesis

**DOI:** 10.1038/sj.bjc.6603539

**Published:** 2007-01-09

**Authors:** R D Verschoyle, P Greaves, H Cai, R E Edwards, W P Steward, A J Gescher

**Affiliations:** 1 Cancer Biomarkers and Prevention Group, Department of Cancer Studies and Molecular Medicine, University of Leicester, Leicester LE2 7LX, UK; 2 Medical Research Council Toxicology Unit, University of Leicester, Leicester, UK

**Keywords:** chemoprevention, diet, rice bran, genetic models of carcinogenesis

## Abstract

Brown rice is a staple dietary constituent in Asia, whereas rice consumed in the Western world is generally white, obtained from brown rice by removal of the bran. We tested the hypothesis that rice bran interferes with development of tumours in TAg, TRansgenic Adenocarcinoma of the Mouse Prostate (TRAMP) or *Apc*^*Min*^ mice, genetic models of mammary, prostate and intestinal carcinogenesis, respectively. Mice received rice bran (30%) in AIN-93G diet throughout their post-weaning lifespan. In TAg and TRAMP mice, rice bran did not affect carcinoma development. In TRAMP or wild-type C57Bl6/J mice, dietary rice bran increased kidney weight by 18 and 20%, respectively. Consumption of rice bran reduced numbers of intestinal adenomas in *Apc*^*Min*^ mice by 51% (*P*<0.01), compared to mice on control diet. In parallel, dietary rice bran decreased intestinal haemorrhage in these mice, as reflected by increased haematocrit. At 10% in the diet, rice bran did not significantly retard *Apc*^*Min*^ adenoma development. Likewise, low-fibre rice bran (30% in the diet) did not affect intestinal carcinogenesis, suggesting that the fibrous constituents of the bran mediate chemopreventive efficacy. The results suggest that rice bran might be beneficially evaluated as a putative chemopreventive intervention in humans with intestinal polyps.

Cancers of the colorectum, breast and prostate constitute major human malignancies for which effective and safe chemoprevention strategies are scarce. Undoubtedly, several recent clinical results validate the conceptual feasibility of cancer chemoprevention in humans. These findings bear out that nonsteroidal anti-inflammatory drugs such as aspirin (Baron *et al*, 2003) or selective inhibitors of cyclooxygenase-2 (COX-2) (Steinbach *et al*, 2000) interfere with colorectal malignancies, that selective oestrogen receptor modulators such as tamoxifen can prevent breast cancer (Fisher *et al*, 1998) and that the *α*-reductase inhibitor finasteride can delay the onset of prostate cancer (Thompson *et al*, 2003). However, chemopreventive interventions using drugs have raised safety concerns. For example, the recent realisation that long-term administration of selective COX-2 inhibitors can detrimentally affect the cardiovascular system, resulting in an increased risk of stroke or cardiac infarction (Fitzgerald, 2004), has dampened the enthusiasm for their extensive use as cancer chemopreventive interventions. Safety problems intrinsic to drugs administered over prolonged periods of time strengthen the interest in diet-related cancer chemoprevention approaches. Epidemiological evidence suggests that human consumption of whole-grain foods may be associated with a low incidence of cancer, especially in the colorectum (Witte *et al*, 1996; World Cancer Research Fund, 1997). Rice, *Oryza sativa*, is the staple food of over half the world's population. The unpolished brown (bran-containing) variety possesses special dietary importance in Asia. Rice consumed in the Western world is generally white and is obtained from brown rice by removal of the bran. Dietary differences such as this may explain why the incidence of cancers, including those of the colorectum, breast and prostate is much lower in Asia than in the Western world (Witte *et al*, 1996). Rice bran contains agents believed to possess cancer chemopreventive properties that are absent from the white variety. Preclinical evidence for anticarcinogenic properties of rice bran, or any of its constituents, is scarce. Promising cancer chemoprevention strategies can suitably be tested in genetic animal models of carcinogenesis by evaluating changes in incidence, multiplicity or volume of preneoplastic and/or neoplastic lesions. In the C3(1) SV40 *T*,*t* antigen transgenic multiple mammary adenocarcinoma (TAg) mouse, a model of breast carcinogenesis, expression of the SV40 transforming sequences (*T* and *t* antigen) is targeted to the mammary epithelium by a fragment of the rat prostatic steroid-binding protein promoter C3(1) (Maroulakou *et al*, 1994). The *T*-antigen binds and functionally inactivates *p53* and *Rb* tumour-suppressor genes (Dyson *et al*, 1989; Mietz *et al*, 1992). The consequent perturbation of cell homeostasis is thought to be responsible for mammary carcinogenesis, and all female TAg mice develop palpable tumours from approximately 12 weeks of age (Maroulakou *et al*, 1994). A similar genetically modified rodent species is the ‘TRansgenic Adenocarcinoma of the Mouse Prostate’ (TRAMP) model, in which expression of the SV40 transforming sequences is targeted to the prostate by a prostate-specific rat probasin promoter (Greenberg *et al*, 1995). All male TRAMP mice develop prostate cancer from approximately 18 weeks of age (Gingrich *et al*, 1999). The *Apc*^*Min*^ mouse is a model of gastrointestinal carcinogenesis genetically driven by a truncating *Apc* gene mutation (Luongo *et al*, 1994), and it resembles the human heritable condition, familial adenomatous polyposis coli (FAP). Among diet-derived agents that have been found to impede carcinogenesis in these models are tea preparations, which interfered with carcinogenesis in TRAMP (Gupta *et al*, 2001) and TAg mice (Kaur, Greaves, Cooke, Edwards, Steward, Gescher and Marczylo, submitted), and the yellow spice curcumin, which compromised adenoma development in *Apc*^*Min*^ mice (Mahmoud *et al*, 2000; Perkins *et al*, 2002). In the light of the putative health benefit, which might be derived from rice bran, we tested the hypothesis that its consumption interferes with breast, prostate or intestinal carcinogenesis in the TAg, TRAMP or *Apc*^*Min*^ mouse models. We found that rice bran reduced adenoma development in the *Apc*^*Min*^ mouse, and dissected the role which the nonfibrous components of the bran preparation may play in mediating chemopreventive activity by studying the effect of low fibre rice bran.

## MATERIALS AND METHODS

### Animals

Breeding colonies were established with: (i) TAg mice on an FVB background, (ii) TRAMP mice on a C57BL/6J background and (iii) C57BL/6J Min/+ (*Apc*^*Min*^) mice. Mice were bred in the Leicester University Biomedical Services facility using animals originally obtained from either the Jackson Laboratory (Bar Harbor, ME, USA, *Apc*^*Min*^ and TAgs) or the NCI Mouse Repository (NCI Frederick Rockville, MD, USA, TRAMP). Ear tissue from newborn mice was genotyped for the presence of the transgene using PCR, as described previously (Maroulakou *et al*, 1994; Perkins *et al*, 2002; The Jackson Laboratory website: www.jax.org).

### Rice bran

Two stabilised rice bran preparations (‘Rice X Stabilized Rice Bran – Regular’ and ‘Rice X Solubles’), produced by the Rice X Comp (El Dorado Hills, CA, USA), were either purchased from Alexander Essentials (Morecambe, UK) or obtained as a gift from the RiceX Comp. ‘Rice X Stabilized Rice Bran – Regular’, referred to in the following as ‘rice bran’, is produced by milling brown rice, a process which releases an active lipase. The milling process includes a ‘stabilisation’ step involving elevated temperature and pressure to ensure lipase deactivation. According to the providers' product data sheet, ‘Rice X Stabilized Rice Bran – Regular’ contains (all values expressed per 100 g rice bran) 29 g dietary fibre, 15 g protein, 21 g fat and 22 g available carbohydrate. This bran preparation contains the following vitamins and minerals: carotenoids (129 *μ*g), vitamin B complex (57 mg), vitamin E complex (26 mg), folic acid (27 *μ*g), biotin (14 *μ*g), choline (105 mg), inositol (1496 mg) *γ*-oryzanol (245 mg), phytosterols (341 mg), sodium (8 mg), potassium (1573 mg), calcium (40 mg), magnesium (727 mg), phosphorus (1591 mg), manganese (26 mg), iron (8 mg) and zinc (6 mg). ‘Rice X Solubles’ (referred to in the following as ‘low-fibre rice bran’) is a powdered emulsion of soluble stabilised rice bran omitting insoluble fibre. It contains (all values per 100 g rice bran) 3 g dietary fibre, 8 g protein, 27 g fat and 55 g available carbohydrate. This bran preparation contains the following vitamins and minerals: carotenoids (47 *μ*g), vitamin B complex (92 mg), vitamin E complex (18 mg), folic acid (37 *μ*g), biotin (15 *μ*g), choline (150 mg), inositol (1314 mg), *γ*-oryzanol (248 mg), phytosterols (413 mg), sodium (16 mg), potassium (1562 mg), calcium (8 mg), magnesium (171 mg), phosphorus (763 mg), manganese (3 mg), iron (2 mg) and zinc (2 mg).

### Animal experiments

Experiments were carried out under animal project licence PPL 40/2496, granted to Leicester University by the UK Home Office. The experimental design was vetted by the Leicester University Local Ethical Committee for Animal Experimentation and met the standards required by the UKCCCR guidelines (Workman *et al*, 1998). Groups of 10–16 mice at 4 weeks of age received standard AIN 93G diet or AIN diet supplemented with rice bran (30 or 10%) to the end of the animals' life. No attempt was made to adjust nutritional components of the AIN93 diet to compensate for the addition of rice bran. The calorific values of AIN 93 G diet or AIN diet containing 30% rice bran or 30% low-fibre rice bran were 377, 363 and 410 calories, respectively, per 100 g diet (based on Reeves *et al*, 1993, and the rice bran provider's product data sheets). Addition of rice bran decreased the protein and carbohydrate content of the overall diet slightly, and increased the fat content from 7% in control AIN diet to 11% for the diet containing 30% rice bran and to 14% for the low-fibre rice bran diet (30%). From 11 weeks of age, TAg and TRAMP mice were palpated once or twice weekly for presence of tumours. TAg tumour size was measured using callipers, and tumour volume was calculated using the equation:*V*=0.5236 × *D* × *d*^2^, with *D* and *d* representing the long and the short diameters, respectively. Animals were killed by cardiac exsanguination (halothane anaesthesia) in weeks 18 (*Apc*^*Min*^), 19 (TAg) or 34 (TRAMP). The intestinal tract of *Apc*^*Min*^ mice was removed and flushed with phosphate-buffered saline. Intestinal tissue was cut open longitudinally and examined under a magnifying lens. Multiplicity, location and size of adenomas were recorded as described before (Perkins *et al*, 2002).

Packed red cell volume (haematocrit) was measured as described before (Strumia *et al*, 1954). Tumours in TAg mice, prostate with prostate tumour plus seminal vesicles, livers, kidneys and lungs of wild-type C57BL6/J or TRAMP mice were excised, weighed and placed in buffered formalin (for histopathology).

### Histopathology

The following tissues were fixed in formalin for a minimum of 2 weeks: the pelt from TAg mice, the intestinal tract from *Apc*^*Min*^ mice, the prostate with prostate tumour plus seminal vesicles and lungs, liver, salivary glands, kidney, spleen, pancreas, gut and dorsal abdominal connective tissue, including inguinal and lumbar lymph nodes from TRAMP mice or wild-type mice. TAg mouse pelts were cut to yield five transverse blocks corresponding to the five pairs of mammary glands. All tissues were embedded in paraffin wax and sections (5 *μ*m thick) were cut and stained with haematoxylin and eosin before microscopic examination. For estimation of numbers of microadenomatous crypts in the colorectum of *Apc*^*Min*^ mice, the formalin-fixed colorectal tract was placed in 0.5% aqueous methylene blue solution (20 s). Excess stain was removed (water), and tissue was flattened between two microscope slides (held in place with elastic bands) and scanned microscopically.

### Statistical evaluation

Evaluation of significance of values, as compared to the appropriate controls, was performed by either one-way analysis of variance with subsequent Tukey's pairwise comparison or a two-sample Student's *t*-test.

## RESULTS

### Effect of rice bran on murine body weight

**Figure 1:**
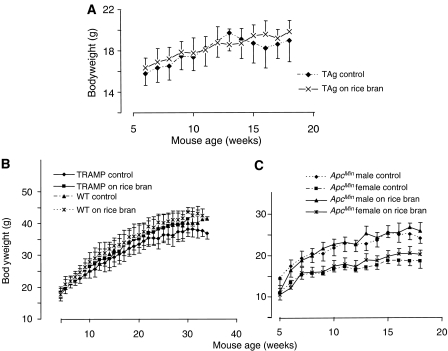
Effect of rice bran on whole body weight of TAg mice (**A**), TRAMP mice and their wild-type (C57Bl/6J) counterparts (**B**) or *Apc*^*Min*^ mice (**C**). Mice received control diet or diet fortified with rice bran at 30%. Results are the mean±s.d. of 12–16 mice. For details of animal experimentation, see Materials and methods.

TAg, TRAMP, their C57Bl/6J wild-type counterparts and *Apc*^*Min*^ mice received rice bran in their diet (30%, ∼0.9 g per mouse=∼36 g kg^−1^ per day) from weaning until the end of the experiment, which was week 18 for the *Apc*^*Min*^ mice, week 19 for the TAg mice and week 34 for the TRAMP mice. Figure 1 shows that the animals' body weight was not significantly different from that of mice on the control diet. There is an indication that TRAMP, wild-type C57Bl/6J and female *Apc*^*Min*^ mice on rice bran were marginally heavier than those on AIN diet alone (Figure 1B, C). Overall, this result suggests that rice bran in the diet does not adversely affect food intake.

### Effect of rice bran on TAg and TRAMP mice

On histopathological investigation, TAg mice presented with intra-duct hyperplasia, intra-duct carcinoma and invasive mammary carcinoma, occasional intra-duct papillomas were also present. In TRAMP mice, proliferative lesions in the prostate formed a continuum between increasing degrees of glandular hyperplasia with atypical cytological features through to frank adenocarcinoma without clearly defined nodular benign neoplasia or adenoma. These observations are consistent with the original description of the TAg and TRAMP mouse models (Maroulakou *et al*, 1994; Gingrich *et al*, 1999). There was no clear difference between control and treated mice with respect to TAg or TRAMP tumour histopathology.

**Figure 2:**
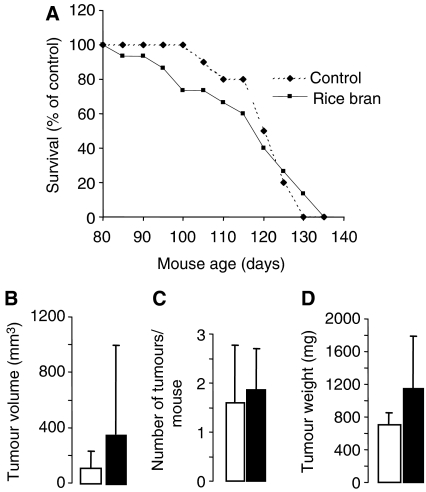
Lack of effect of rice bran on mammary carcinogenesis in TAg mice as reflected by survival (**A**), tumour volume (**B**), tumour multiplicity (**C**) and tumour weight (**D**). Mice received control diet (open bars) or 30% rice bran in the diet (closed bars) from week 3 after weaning for their lifetime. Animals were killed when tumour diameter exceeded 17 mm. Volume, multiplicity and weights of tumours were determined at the termination of the experiment. Results are the mean±s.d. (*n*=12 for controls and 15 for intervention group). For details of experimental design and assessment of TAg tumour development see Materials and methods.

**Figure 3:**
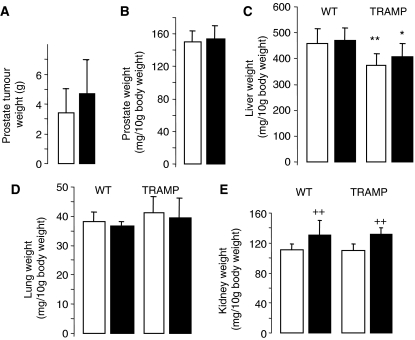
Effect of rice bran on weight of the following tissues in TRAMP mice (**A**, **C**–**E**) or C57Bl/6J (wild-type) mice (**B**–**E**): prostate carcinoma plus seminal vesicles (**A**), normal prostate plus seminal vesicles (**B**), liver (**C**), lung (**D**) and kidney (**E**). Mice received control diet (open bars) or diet fortified with 30% rice bran (closed bars) from weaning for their lifetime. Tissue weight was determined at the termination of the experiment. Results are the mean±s.d. of 12–16 mice. Stars indicate that liver weight in TRAMP mice was significantly lower than that in wild-type mice (^*^P<0.02, ^**^P<0.005), and crosses indicate that kidney weights in mice on rice bran were significantly higher than those in mice on control diet (^++^*P*<0.002). For details of experimental design and assessment of tissue weight, see Materials and Methods.

Consumption of bran failed to significantly affect mammary carcinogenesis in TAg mice, as reflected by survival of animals (Figure 2A), tumour volume (Figure 2B), number of tumours per mouse (Figure 2C) or tumour weight at the end of the experiment (Figure 2D). The results shown in Figure 2B and D tentatively hint at slightly increased tumour weight and volume in TAg mice on rice bran compared to controls. Rice bran consumption did not interfere with prostate carcinogenesis in TRAMP mice, as mirrored by tumour weight at the end of the experiment (Figure 3A).

Healthy prostate, liver, kidney, lung spleen, lymph nodes, pancreas and gut tissues in TRAMP and wild-type C57Bl/6J mice were closely inspected for potential effects of rice bran. The weight of healthy prostate in C57BL/6J (Figure 3B) and of liver and lung in wild-type C57BL/6J or TRAMP mice (Figure 3C and D) was not affected by rice bran consumption. Livers in TRAMP mice weighed significantly less than those in wild-type mice, irrespective of diet (Figure 3C). For mice on control diet, the difference was 18% and for mice on rice bran 13%. These livers showed variable degree of clear cell change (glycogen) and vacuolation (fat) typical of well-fed mice without clear histological differences between rice bran-fed and control mice. Kidneys in wild-type or TRAMP mice that received 30% rice bran weighed 20 or 18%, respectively, more than kidneys in mice on control diet (Figure 3E). On histopathological inspection, large clear vacuoles, indicative of lipid droplets without evidence of cellular degeneration, were present in the proximal tubular cell cytoplasm in all mice, albeit more prominently in the mice that received rice bran.

### Effect of rice bran on intestinal adenoma development in *Apc*^*Min*^ mice

**Figure 4:**
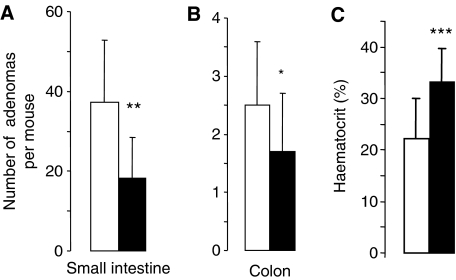
Effect of 30% dietary rice bran on adenoma number in the small intestine (**A**) or colon (**B**) and on haematocrit (**C**) in *Apc*^*Min*^ mice. Mice received control diet (open bars) or diet fortified with 30% rice bran (closed bars) from week 3 after weaning for their lifetime. Results are the mean±s.d., *n*=15. Stars indicate that value is significantly different from control (^*^P<0.05, ^**^P<0.01, ^***^P<0.001). For details of experimental design and assessment of adenoma number, see Materials and Methods.

**Figure 5:**
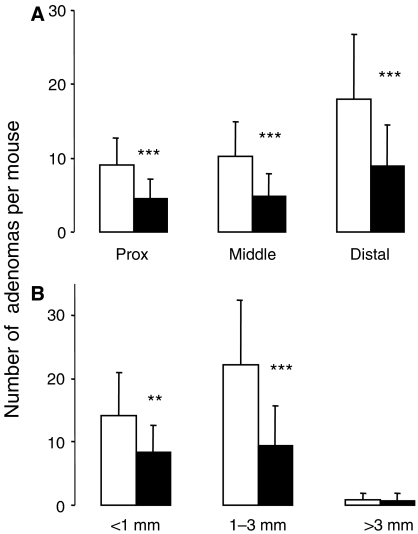
Effect of dietary 30% dietary rice bran on adenoma multiplicity in the proximal (‘prox’), middle and distal sections of the small intestine (**A**), and on total multiplicity of small (<1 mm diameter), medium-sized (1–3 mm) or large (>3 mm) adenomas (**B**) in *Apc*^*Min*^ mice. Mice received control diet (open bars) or diet containing 30% rice bran (closed bars) from week 3 after weaning for their lifetime. Results, which are presented as number of adenomas per mouse as related to distribution (**A**) or size (**B**), are the mean±s.d. (*n*=15). Stars indicate that values are significantly different from controls (^*^P<0.05, ^**^P<0.01, ^***^P<0.005). For details of experimental design and assessment of adenoma number, see Materials and Methods.

**Figure 6:**
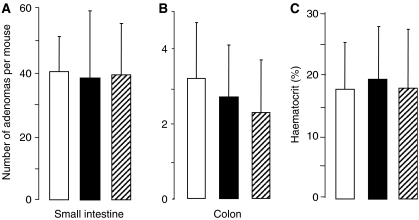
Lack of effect of 10% dietary rice bran (closed bars) or 30% dietary low-fibre rice bran (crossed bars) on adenoma number in the small intestine (**A**) or colon (**B**) and on haematocrit (**C**) in *Apc*^*Min*^ mice. Mice received control diet (open bars) or diet fortified with 10% rice bran (closed bars) or 30% low-fibre rice bran (crossed bars) from week 3 after weaning for their lifetime. Results are the mean±s.d., *n*=16–17. For details of experimental design and assessment of adenoma number, see Materials and Methods.

Histopathological analysis of the small intestine of *Apc*^*Min*^ mice, which had received control diet or rice bran (30%) during their life time, showed focal proliferative lesions ranging from hyperplastic glands to larger areas of glandular hyperplasia and polypoid adenomas. There were no significant differences in tumour morphology in terms of dysplasia between control mice and mice on rice bran, which suggests that there was no difference in tumour aggressiveness. The numbers of adenomas in the small intestine or colon of mice on rice bran were significantly reduced by 51 and 32%, respectively, compared to mice on control diet (Figure 4A and B). A detailed analysis of small intestinal polyp location revealed that polyp numbers in the proximal, middle and distal sections of the intestine were similarly affected by rice bran (Figure 5A). The effect of bran on adenoma development was more notable in small and medium-sized polyps (<3 mm) than in large ones (>3 mm) (Figure 5B), even though the overall small number of large polyps observed may have obfuscated any difference. At the late stage of adenoma development, *Apc*^*Min*^ mice suffer from intestinal bleeding, which causes a dramatic fall in haematocrit. Intervention with rice bran raised the haematocrit measured at the end of the experiment, from 22.2% in untreated *Apc*^*Min*^ mice to 33.1%, consistent with impeded adenoma development (Figure 4C). In order to test the hypothesis that a smaller dose of rice bran may affect adenoma development, the experiment was repeated by including mice on 10% dietary rice bran. In this repeat experiment, rice bran at 30% reduced small intestinal adenoma load by 45% (from 40±11 per mouse to 22±15, mean±s.d., *n*=15–17, *P*<0.005) and colonic adenomas by 31% (from 3.2±1.5 to 2.2±1.1, *P*<0.05), in accordance with the results shown in Figure 4. In contrast, small intestinal or colonic adenoma numbers were not significantly diminished by 10% rice bran (Figure 6A and B). Consistent with these results, the haematocrit in *Apc*^*Min*^ mice on 30% rice bran (28.6±9.9%) was significantly higher than that in mice on the control diet (17.7±7.6%, *P*=0.002), whereas 10% rice bran did not significantly increase the haematocrit compared to controls (Figure 6C).

In order to explore whether nonfibrous-constituents of rice bran mediate the retardation of adenoma development, *Apc*^*Min*^ mice received a low-fibre rice bran preparation with their diet at the same dose (30%), at which high-fibre rice bran reduced adenomas. The numbers of adenomas in the small intestine and colon of mice on low-fibre rice bran were not significantly different from those in mice on control diet (Figure 6A and B), and the haematocrit reflected the lack of effect of low-fibre rice bran on adenoma development (Figure 6C). Thus, in contrast to high-fibre-containing rice bran, low-fibre rice bran failed to affect adenoma formation.

It has been suggested that numbers of microadenomatous crypts in the colorectal tract of *Apc*^*Min*^ mice might allow a judgment to be made as to the effect of potential chemopreventive interventions, in a fashion arguably more relevant to the human disease counterpart than by counting adenomas in the small intestine (Yamada *et al*, 2002). When we enumerated the microadenomatous crypts identifiable in the colorecta of *Apc*^*Min*^ mice in this experiment, 16 of the 51 mice (31%) had a total of 37 microadenomatous crypts. Individually, the mice displayed between one and seven lesions, far fewer than the >20 lesions per mouse observed by Yamada *et al* (2002). There was no difference in propensity between mice on control diet and those on rice bran to bear microadenomatous crypts.

## DISCUSSION

The results presented here suggest for the first time, that while rice bran possesses cancer chemopreventive efficacy in the *Apc*^*Min*^ mouse model of colorectal carcinogenesis, it lacks anticarcinogenic activity in the TAg or TRAMP mouse models of mammary and prostate cancer. The fat content of the rice bran preparation may have caused the observed slight elevation compared to controls of TAg mouse tumour weight and volume; and of bodyweight of TRAMP, wild-type C57Bl6/J and female *Apc*^*Min*^ mice. The results also suggest that the adenoma-retarding activity of rice bran was dose-related, that it was exerted evenly along all sections of the murine intestinal tract, and that activity was associated predominantly with the fibre content of the bran rather than the nonfibrous constituents. These results, obtained in three genetic carcinogenesis models, contribute to the emerging preclinical evidence that brown rice products possess cancer chemopreventive properties in rodents. Evidence for potential benefit seems to accumulate especially in colorectal carcinogenesis models. Rice germ (2.5% in the diet), and Kurosu, a vinegar generated from unpolished rice, prevented azoxymethane-induced colon carcinogenesis in rats (Kawabata *et al*, 1999; Shimoji *et al*, 2004). A brown rice preparation (2.5 or 5% in the diet), obtained by fermentation with *Aspergillus oryzae*, interfered with azoxymethane-induced formation of aberrant crypt foci and adenocarcinomas in rats (Katayama *et al*, 2002). Furthermore, fermented brown rice reduced diethylnitrosamine- and phenobarbital-induced hepatocarcinogenesis in rats (Katayama *et al*, 2003), *N*-nitrosomethylbenzylamine-induced oesophageal tumorigenesis in rats (Kuno *et al*, 2004) and bladder carcinogenesis in mice (Kuno *et al*, 2006). To our knowledge there is no information in the literature on the epidemiology, linking consumption of brown rice or rice bran to colorectal cancer risk in humans, which would complement the available data in animal models.

The result described here in the *Apc*^*Min*^ mouse is consistent with reports on the effect of rye bran (10% in the diet administered for 5–6 weeks, Mutanen *et al*, 2000) and wheat bran (5–20% for 7–9 weeks, Hioki *et al*, 1997; Yu *et al*, 2001), which have previously been shown to reduce adenoma development in this model. Molecules in the rice bran fibre fraction, which may have contributed to inhibition of adenoma formation observed here in the *Apc*^*Min*^ mouse, are the carbohydrates dextran-like *α*-glucan and arabinoxylan hemicellulose, both of which have previously been suggested to possess cancer chemopreventive properties (Takeo *et al*, 1988; Takenaka and Takahashi, 1991; Aoe *et al*, 1993). In principle there are three types of mechanism by which dietary fibre is thought to interfere with colorectal carcinogenesis (for review, see Young *et al*, 2005), and they may also be germane to rice bran fibre. These mechanisms have been the subject of much investigation, although details and their relative importance are unresolved. Firstly, fibre is thought to exert ‘physical’ effects such as increasing faecal bulk, hastening faecal transit and binding potentially co-carcinogenic bile salts. Secondly, fibre can change the gastrointestinal microflora and luminal environment in such a way as to alter bacterial species, that may, in turn, reduce bile salt metabolism. Thirdly, fibre carbohydrates can undergo fermentation in the luminal environment, generating short chain fatty acids such as butyrate, which are thought to exert anticarcinogenic effects such as inhibition of cell proliferation and induction of differentiation and apoptosis. The lack of effect of rice bran on carcinogenesis, in the genetic models of mammary and prostate cancer described here, is consistent with the fact that it was the fibre fraction of the bran which mediated the anticarcinogenic effects in the *Apc*^*Min*^ mouse, and that fibre components would be unlikely to reach organs remote from the gastrointestinal tract.

The rice bran dose in humans that would be equivalent to the 30% dietary dose (∼36 g kg^−1^ *pd*), which was active in the mice described here, when calculated on the basis of equivalent body surface area, would be 115 g m^−2^ or 207 g per person *pd*, assuming a body surface area of 1.8 m^2^ accompanying a body weight of 70 kg (Freireich *et al*, 1966). Although this is a hefty dose, it is feasible and very probably safe. In our study, a preliminary investigation of effects of 30% dietary rice bran on the murine organism, as reflected by weights of the whole body, liver, lung, kidney or prostate, did not uncover major untoward effects. The only unusual effect was a small increase in kidney weight associated with a slight increase in tubular lipid, content but without associated degenerative alterations. We also discovered that livers in TRAMP mice are slightly but significantly smaller than those in their wild-type counterparts, a phenomenon that has not been reported thus far. One may speculate that prostate tumours secrete agents that specifically retard liver weight gain.

Correlations between rodent and human data are imperfect with respect to chemopreventive efficacy and safety. Nevertheless, a retrospective analysis of results in rodents and humans, obtained with inhibitors of COX enzymes in colorectal cancer prevention, shows reasonable consistency (Hawk and Levin, 2005). The *Apc*^*Min*^ mouse model predicted the adenoma-regressing activity of the NSAID sulindac and the COX-2 inhibitor celecoxib (Boolbol *et al*, 1996; Jacoby *et al*, 2000) in familial adenomatous polyposis coli patients (Giardiello *et al*, 1993; Steinbach *et al*, 2000). Unwanted side effects of NSAIDs and of COX-2 inhibitors, the latter of which have recently received considerable attention (Fitzgerald, 2004), may ultimately militate against their extensive use as cancer chemopreventive agents in humans. Therefore, the search for toxicologically innocuous alternative interventions is timely and propitious, and foodstuffs provide an attractive focus for this search. In the light of the safety of rice bran and its efficacy in the *Apc*^*Min*^ mouse described here, as well as in carcinogen-induced rat models of colorectal cancer (Kawabata *et al*, 1999; Katayama *et al*, 2002; Shimoji *et al*, 2004), we suggest that rice bran might be worthy of consideration for clinical evaluation as an intervention to prevent adenoma recurrence. In contrast, our results in two genetic models of mammary and prostate cancer do not support the inclusion of rice bran in the portfolio of candidate interventions for clinical development in the chemoprevention of breast or prostate cancer.
